# Facial recognition technology can expose political orientation from naturalistic facial images

**DOI:** 10.1038/s41598-020-79310-1

**Published:** 2021-01-11

**Authors:** Michal Kosinski

**Affiliations:** Stanford University, Stanford, CA 94305 USA

**Keywords:** Psychology, Information technology, Computer science

## Abstract

Ubiquitous facial recognition technology can expose individuals’ political orientation, as faces of liberals and conservatives consistently differ. A facial recognition algorithm was applied to naturalistic images of 1,085,795 individuals to predict their political orientation by comparing their similarity to faces of liberal and conservative others. Political orientation was correctly classified in 72% of liberal–conservative face pairs, remarkably better than chance (50%), human accuracy (55%), or one afforded by a 100-item personality questionnaire (66%). Accuracy was similar across countries (the U.S., Canada, and the UK), environments (Facebook and dating websites), and when comparing faces across samples. Accuracy remained high (69%) even when controlling for age, gender, and ethnicity. Given the widespread use of facial recognition, our findings have critical implications for the protection of privacy and civil liberties.

## Introduction

There is a growing concern that the widespread use of facial recognition will lead to the dramatic decline of privacy and civil liberties^1^. Ubiquitous CCTV cameras and giant databases of facial images, ranging from public social network profiles to national ID card registers, make it alarmingly easy to identify individuals, as well as track their location and social interactions. Moreover, unlike many other biometric systems, facial recognition can be used without subjects’ consent or knowledge.

Pervasive surveillance is not the only risk brought about by facial recognition. Apart from identifying *individuals*, the algorithms can identify individuals’ *personal attributes*, as some of them are linked with facial appearance. Like humans, facial recognition algorithms can accurately infer gender, age, ethnicity, or emotional state^2,3^. Unfortunately, the list of personal attributes that can be inferred from the face extends well beyond those few obvious examples.

A growing number of studies claim to demonstrate that people can make face-based judgments of honesty^4^, personality^5^, intelligence^6^, sexual orientation^7^, political orientation^8–12^, and violent tendencies^13^. There is an ongoing discussion about the extent to which face-based judgments are enabled by stable facial features (e.g., morphology); transient facial features (e.g., facial expression, makeup, facial hair, or head orientation); or targets’ demographic traits that can be easily inferred from their face (e.g., age, gender, and ethnicity)^14^. Moreover, the accuracy of the human judgment is relatively low. For example, when asked to distinguish between two faces—one conservative and one liberal—people are correct about 55% of the time (derived from Cohen’s d reported in Tskhay and Rule^15^), only slightly above chance (50%). Yet, as humans may be missing or misinterpreting some of the cues, their low accuracy does not necessarily represent the limit of what algorithms could achieve. Algorithms excel at recognizing patterns in huge datasets that no human could ever process^16^, and are increasingly outperforming us in visual tasks ranging from diagnosing skin cancer^17^ to facial recognition^18^ to face-based judgments of intimate attributes, such as sexual orientation (76% vs. 56%)^7,19^, personality (64% vs. 57%; derived from Pearson’s r*s*)^20–22^, and—as shown here—*political orientation*. (For ease of interpretation and comparisons across studies, across this text, accuracy is expressed as the area under the receiver operating characteristic curve (AUC), an equivalent of the Wilcoxon signed-rank test coefficient and the common language effect size.)

## Methods

We used a sample of 1,085,795 participants from three countries (the U.S., the UK, and Canada; see Table 1) and their self-reported political orientation, age, and gender. Their facial images (one per person) were obtained from their profiles on Facebook or a popular dating website. These self-selected, naturalistic images combine many potential cues to political orientation, ranging from facial expression and self-presentation to facial morphology. The ethnic diversity of our sample (it included over 347,000 non-white participants), the relative universality of the conservative–liberal spectrum^23^, and the generic type of facial images used here increase the likelihood that our findings apply to other countries, cultures, and types of images.

**Table 1:** Number of participants and the distribution of political orientation, gender, age, and ethnicity.

Dataset	Country	Political orientation	Number of participants	% Female	% White	Age	
						Median	IQR
Dating website	U.S.	Conservative	463,367	60%	72%	41	[31,53]
		Liberal	399,403	72%	64%	38	[31,50)
	Canada	Conservative	23,407	52%	73%	38	[30,53]
		Liberal	43,715	67%	69%	37	[32,50]
	UK	Conservative	19,604	52%	78%	39	[31,49]
		Liberal	28,281	58%	73%	36	[30,43)
Facebook	U.S.	Conservative	40,905	62%	73%	27	[24,35]
		Liberal	67,113	66%	55%	27	[24,34]
		Total:	1,085,795	65%	68%	37	[29,50]

IQR stands for interquartile range.

**Figure 1:**
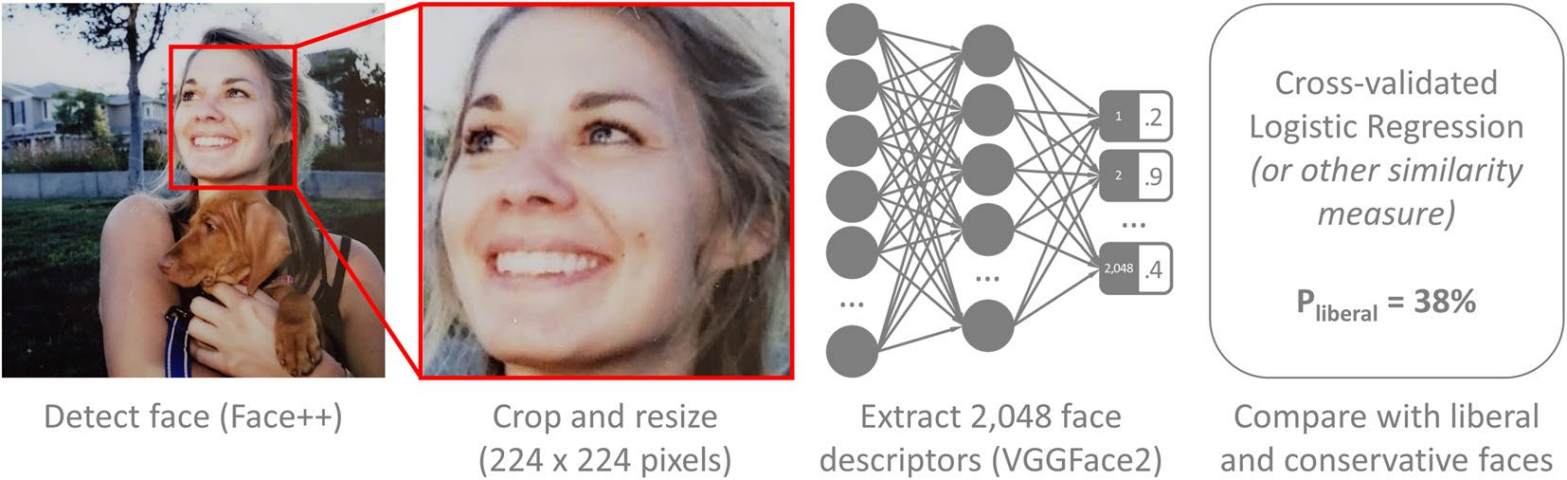
Procedure used to predict political orientation from facial images. (To protect participants’ privacy, we used a photo of a professional model. Their informed consent for publication was obtained.)

As we are aiming to study existing privacy threats, rather than develop new privacy-invading tools, we used an open-source facial-recognition algorithm instead of developing an algorithm specifically aimed at political orientation. The procedure is presented in Fig. 1: To minimize the role of the background and non-facial features, images were tightly cropped around the face and resized to 224 × 224 pixels. VGGFace2^24^ was used to convert facial images into *face descriptors*, or 2,048-value-long vectors subsuming their core features. Usually, similarity between face descriptors is used to identify those similar enough to likely represent the face of the same person. Here, to identify individuals’ political orientation, their face descriptors are compared with the average face descriptors of liberals versus conservatives. Descriptors were entered into a cross-validated logistic regression model aimed at self-reported political orientation (conservative vs. liberal). Virtually identical results were produced by alternative methods: a deep neural network classifier and a simple ratio between average cosine similarity to liberals and conservatives. See the Supplementary Methods section for more details.

## Results

**Figure 2:**
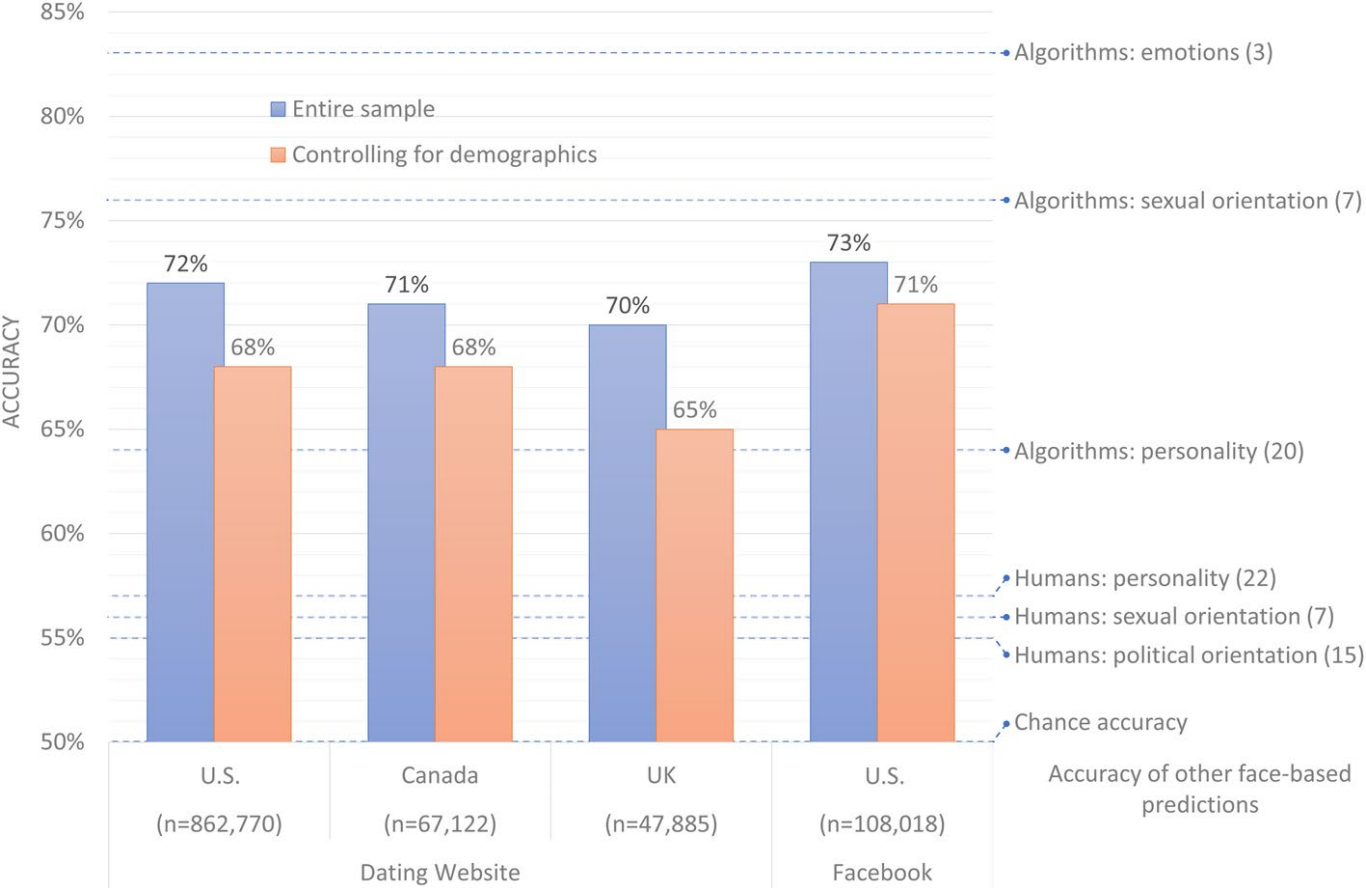
Accuracy of the facial-recognition algorithm predicting political orientation. All 95% confidence intervals are below 1% and are thus omitted. Humans’ and algorithms’ accuracy reported in other studies is included for context.

The results are presented in Fig. 2 (blue bars). The accuracy is expressed as AUC, or a fraction of correct guesses when distinguishing between all possible pairs of faces—one conservative and one liberal. In the largest sample, of 862,770 U.S. dating website users, the cross-validated classification accuracy was 72%, which is much higher than chance (50%) and translates into Cohen’s d = 0.83, or a *large* effect size. (Sawilowsky^25^ suggested the following heuristic for interpreting effect sizes: very small [d ≥ 0.01], small [d ≥ 0.2], medium [d ≥ 0.5], large [d ≥ 0.8], very large [d ≥ 1.2], and huge [d ≥ 2].) Similar accuracy was achieved for dating website users in Canada (71%) and in the UK (70%). The predictability was not limited to the online dating environment: The algorithm’s accuracy reached 73% among U.S. Facebook users. To put the algorithm’s accuracy into perspective, consider that human accuracy in similar tasks is 55%, only slightly above chance (SD = 4%; CI_95%_ = [52%,58%])^15^.

Moreover, as shown in Table 2, the algorithm could successfully predict political orientation across countries and samples. Regression trained on the U.S. dating website users, for example, could distinguish between liberal and conservative dating website users in Canada (68%), the UK (67%), and in the Facebook sample (71%). Overall, the average out-of-sample accuracy was 68%, indicating that there is a significant overlap in the links between facial cues and political orientation across the samples and countries examined here.

**Table 2:** Classification accuracy across the subsamples (rows) and models trained on each of the samples (columns). All 95% confidence intervals are below 1% and are thus omitted.

	Dataset	Accuracy (AUC) of models trained on:			
		1	2	3	4
1	Dating website (U.S.)	**72%**	68%	65%	69%
2	Dating website (Canada)	68%	**71%**	63%	65%
3	Dating website (UK)	67%	65%	**70%**	66%
4	Facebook (U.S.)	71%	66%	65%	**73%**

Cross-validated in-sample prediction accuracy is presented in bold.

Both in real life and in our sample, the classification of political orientation is to some extent enabled by demographic traits clearly displayed on participants’ faces. For example, as evidenced in literature^26^ and Table 1, in the U.S., white people, older people, and males are more likely to be conservatives. What would an algorithm’s accuracy be when distinguishing between faces of people of the same age, gender, and ethnicity? To answer this question, classification accuracies were recomputed using only face pairs of the same age, gender, and ethnicity.

The results are presented in Fig. 2 (red bars). The accuracy dropped by only 3.5% on average, reaching 68%, 68%, 65%, and 71% for the U.S., Canadian, and UK dating website users, as well as for the U.S. Facebook users, respectively. This indicates that faces contain many more cues to political orientation than just age, gender, and ethnicity.

Another factor affecting classification accuracy is the quality of the political orientation estimates. While the dichotomous representation used here (i.e., conservative vs. liberal) is widely used in the literature, it offers only a crude estimate of the complex interpersonal differences in ideology. Moreover, self-reported political labels suffer from the reference group effect: respondents’ tendency to assess their traits in the context of the salient comparison group. Thus, a self-described “liberal” from conservative Mississippi could well consider themselves “conservative” if they lived in liberal Massachusetts. Had the political orientation estimates been more precise (i.e., had less error), the accuracy of the face-based algorithm could have been higher. Consequently, apart from considering the absolute classification accuracy, it is useful to compare it with one offered by alternative ways of predicting political orientation. Here, we use personality, a psychological construct closely associated with, and often used to approximate, political orientation^27^. Facebook users’ scores on a well-established 100-item-long five-factor model of personality questionnaire^28^ were entered into a tenfold cross-validated logistic regression to predict political orientation.

**Figure 3:**
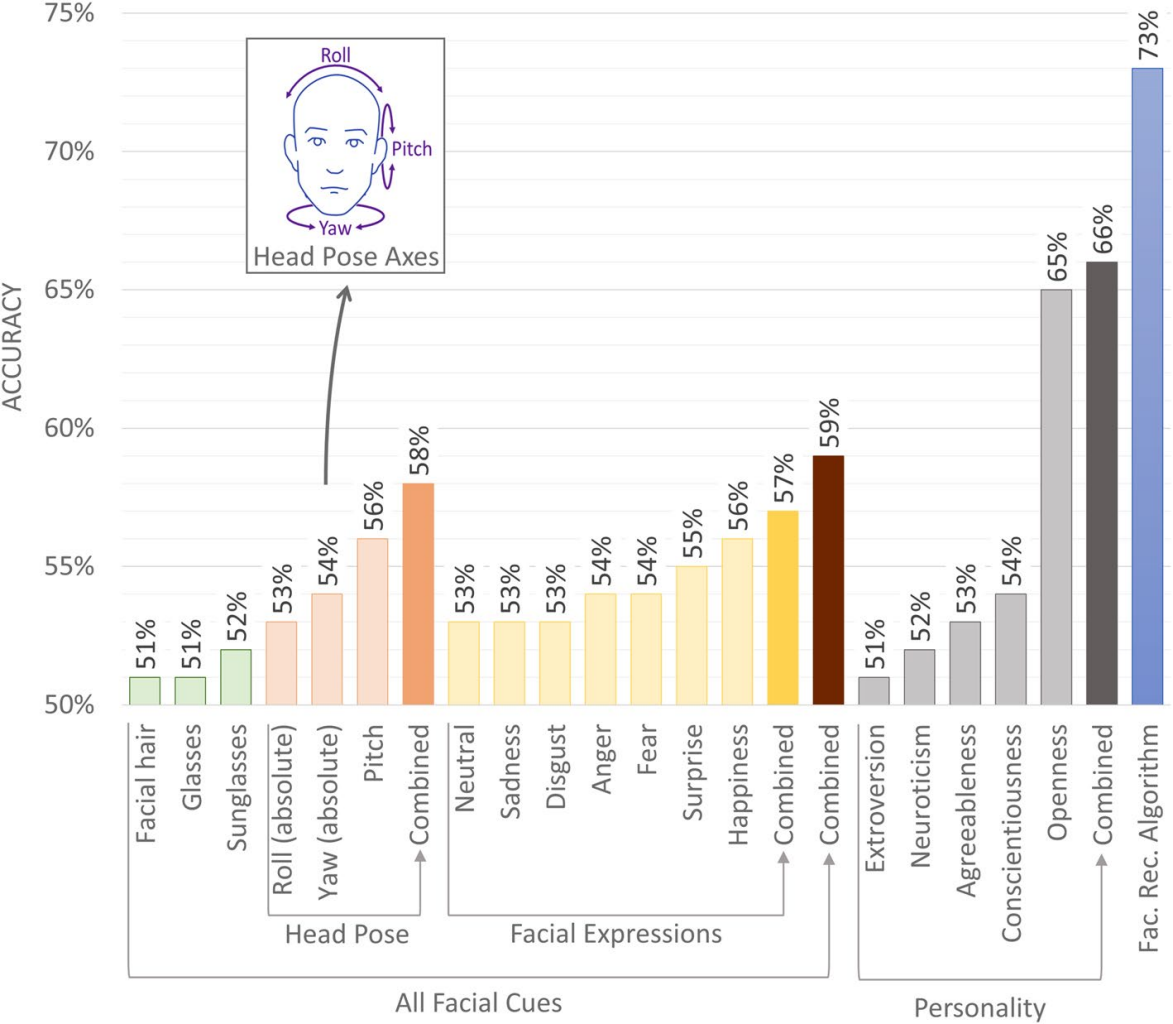
Accuracy afforded by transient facial features and personality traits when predicting political orientation in Facebook users (similar results were obtained in other samples; see Supplementary Table S1). All 95% confidence intervals are below 1% and are thus omitted.

The results presented in Fig. 3 show that the highest predictive power was offered by openness to experience (65%), followed by conscientiousness (54%) and other traits. In agreement with previous studies^27^, liberals were more open to experience and somewhat less conscientious. Combined, five personality factors predicted political orientation with 66% accuracy—significantly less than what was achieved by the face-based classifier in the same sample (73%). In other words, a single facial image reveals more about a person’s political orientation than their responses to a fairly long personality questionnaire, including many items ostensibly related to political orientation (e.g., “I treat all people equally” or “I believe that too much tax money goes to support artists”).

High predictability of political orientation from facial images implies that there are significant differences between the facial images of conservatives and liberals. High out-of-sample accuracy suggests that some of them may be widespread (at least within samples used here). Here, we explore correlations between political orientation and a range of interpretable facial features including head pose (pitch, roll, and yaw; see Fig. 3); emotional expression (probability of expressing sadness, disgust, anger, surprise, and fear); eyewear (wearing glasses or sunglasses); and facial hair. Those features were extracted from facial images and entered (separately and in sets) into tenfold cross-validated logistic regression to predict political orientation.

The results presented in Fig. 3 are based on Facebook users (similar results were obtained in other samples; see Supplementary Table S1). The highest predictive power was afforded by head orientation (58%), followed by emotional expression (57%). Liberals tended to face the camera more directly, were more likely to express surprise, and less likely to express disgust. Facial hair and eyewear predicted political orientation with minimal accuracy (51–52%). Even when combined, interpretable facial features afforded an accuracy of merely 59%, much lower than one achieved by the facial recognition algorithm in the same sample (73%), indicating that the latter employed many more features than those extracted here. A more detailed picture could be obtained by exploring the links between political orientation and facial features extracted from images taken in a standardized setting while controlling for facial hair, grooming, facial expression, and head orientation.

## Discussion

An algorithm’s ability to predict our personal attributes from facial images could improve human–technology interactions by enabling machines to identify our age or emotional state and adjust their behavior accordingly. Yet, the same algorithms can accurately predict much more sensitive attributes, such as sexual orientation^7^, personality^20^ or, as we show here, political orientation. Moreover, while many other digital footprints are revealing of political orientation and other intimate traits^29–34^, one’s face is particularly difficult to hide in both interpersonal interactions and digital records. Facial images can be easily (and covertly) taken by a law enforcement official or obtained from digital or traditional archives, including social networks, dating platforms, photo-sharing websites, and government databases. They are often easily accessible; Facebook and LinkedIn profile pictures, for instance, are public by default and can be accessed by anyone without a person’s consent or knowledge. Thus, the privacy threats posed by facial recognition technology are, in many ways, unprecedented.

Predictability of political orientation from facial images does not necessarily imply that liberals and conservatives have innately different faces. While facial expression or head pose, facial hair, and eyewear were not particularly strongly linked with political orientation in this study, it is possible that a broader range of higher-quality estimates of those and other transient features could fully account for the predictability of political orientation. Yet, from the privacy protection standpoint, the distinction between innate and transient facial features matters relatively little. Consistently changing one’s facial expressions or head orientation would be challenging, even if one knew exactly which of their transient facial features reveal their political orientation. Moreover, the algorithms would likely quickly learn how to extract relevant information from other features—an arms race that humans are unlikely to win.

Some may doubt whether the accuracies reported here are high enough to cause concern. Yet, our estimates unlikely constitute an upper limit of what is possible. Higher accuracy would likely be enabled by using multiple images per person; using images of a higher resolution; training custom neural networks aimed specifically at political orientation; or including non-facial cues such as hairstyle, clothing, headwear, or image background. Moreover, progress in computer vision and artificial intelligence is unlikely to slow down anytime soon. Finally, even modestly accurate predictions can have tremendous impact when applied to large populations in high-stakes contexts, such as elections. For example, even a crude estimate of an audience’s psychological traits can drastically boost the efficiency of mass persuasion^35^. We hope that scholars, policymakers, engineers, and citizens will take notice.

## Supplementary methods

The study has been reviewed and approved by Stanford University’s IRB. All methods were carried out in accordance with relevant guidelines and regulations. The preregistration documents can be found at https://osf.io/y5wr9. The author’s notes are available at: https://bit.ly/kosinski1.

### Dating website sample

The dating website sample was provided by a popular dating website in 2017. It contains profile images uploaded by 977,777 users; their location (country); and self-reported political orientation, gender, and age.

Political orientation was measured using a multiple-choice item. Those who selected “Very conservative” (7%) or “Conservative” (20%) were labeled as conservative; those who selected “Very liberal” (7%) or “Liberal” (16%) were labeled as liberal. Those who selected “Green” (1%), “Libertarian” (2%), “Other” (5%), “Centrist” (17%), or did not know (26%) are not included in this sample. (Those response options are reported in a slightly altered form to protect the identity of the data source.)

Given that people prefer partners of similar political orientation^36^, there should be little incentive to misrepresent one’s views in the context of a dating website. The validity of this variable is also supported by the high accuracy and high external validity of the political orientation classifier.

### Facebook sample

The Facebook sample included public profile images, age, gender, political orientation, and personality scores volunteered by 108,018 U.S. Facebook users recruited through an online personality questionnaire between 2007 and 2012. Participants were rewarded by the feedback on their scores and provided informed consent for their data to be recorded and used in research.

Participants’ personality was measured using the 100-item International Personality Item Pool (IPIP) five-factor model of personality questionnaire^28^, with a five-point Likert-style response scale ranging from “strongly disagree” to “strongly agree.” The scales’ Cronbach’s α reliability equaled 0.84, 0.92, 0.93, 0.88, and 0.93 for openness, conscientiousness, extraversion, agreeableness, and neuroticism, respectively. Two items measuring openness were excluded from scoring because they were used to measure participants’ political orientation (see below).

Participants’ political orientation was established using the “political views” profile field and two items from the IPIP personality questionnaire: “I tend to vote for liberal political candidates” and “I tend to vote for conservative political candidates.” To be labeled as conservative, participants needed to self-describe as “republican” (10%), “conservative” (12%), or “very conservative” (2%); *and* disagree (11%) or strongly disagree (9%) with the former IPIP item; *and* agree (10%) or strongly agree (12%) with the latter. To be labeled as liberal, participants needed to self-describe as “democrat” (15%), “liberal” (15%), or “very liberal” (5%); *and* agree (16%) or strongly agree (26%) with the first IPIP item; *and* disagree (20%) or strongly disagree (17%) with the latter. Participants that did not meet those criteria were not included in this sample.

### Facial images

Facial images were processed using Face++^37^ to detect faces. Images were cropped around the face-box provided by Face++ (red frame on Fig. 1**)** and resized to 224 × 224 pixels. Images with multiple faces, or a face-box narrower than 70 pixels, are not included in our sample.

Additionally, Face++ was used to identify participants’ age, gender, and ethnicity (white, black, East Asian, and Indian); head orientation (pitch, roll, and yaw; see Fig. 3); emotional expression (probability of expressing sadness, disgust, anger, surprise, and fear); and the presence of any kind of glasses or sunglasses. The accuracy of these estimates was good. Predicted and self-reported age correlated at the R = 0.65 level (*p* < 0.001; root mean square error equaled three years). Predicted and self-reported gender matched for 96% of participants. Ethnicity estimated by Face++ and a hypothesis-blind research assistant matched for 88% of 27,023 facial images, a subset of the Facebook sample. Two hypothesis-blind research assistants labeled a subset of 300 images from the Facebook sample with estimates of facial expression and head pose. The correlation between their averaged rankings and Face++ estimates was r = 0.72 on average (see Supplementary Table S2).

### Classification algorithm

Classification employed L2-normalized face descriptors derived from facial images using the VGGFace2 model in ResNet-50 architecture, originally trained on an independent sample of over 3.3 million facial images^24^. We tested three approaches to measuring faces’ relative similarity to faces of liberal and conservative others:

- *Cosine similarity ratio:* For each face, we took the ratio between its average cosine similarity with liberal faces and between its average cosine similarity with conservative faces.
- *Logistic regression:* Face descriptors were entered into LASSO logistic regression^38^ aimed at distinguishing between liberals and conservatives. We used a 30-fold cross-validation so that predictions were made by classifiers that have not seen a given participant before. Parameter α was set to 1; parameter δ was fitted separately within each training set using tenfold cross-validation.
- *Neural Networks:* Face descriptors were entered into a 30-fold cross-validated deep neural network aimed at classifying liberals and conservatives. We tested several network architectures, yet the accuracy did not substantially exceed one offered by two previous, much simpler, approaches.

Given that all three methods yielded similar classification accuracies, we decided to employ LASSO logistic regression. It is computationally efficient and well known among social scientists.

### Classification accuracy

Classification accuracy is expressed as AUC. Red bars in Fig. 2 represent the accuracy estimated on the conservative–liberal face pairs of the same age (+ /− one year), gender, and ethnicity. We employed Face++ estimates of these traits, as they were available for all faces. Similar accuracy (71%) was achieved when using ethnicity labels produced by a research assistant and self-reported age and gender (ethnicity labels were available for a subset of 27,023 images in the Facebook sample).

### Facial hair classifier

Facial hair classifier was built using VGGFace2 face descriptors. A hypothesis-blind research assistant labeled 10,000 facial images of males for the presence of facial hair (dichotomous variable) from the Facebook sample. A second hypothesis-blind research assistant labeled a subset of 2,000 of these images: The inter-rater agreement equaled 95%. Facial hair was present on 59% of faces.

These manual labels were used to train LASSO logistic regression^38^, employing face descriptors to estimate the probability of a given face to contain facial hair. Parameter α was set to 1; parameter δ was fitted using tenfold cross-validation. The tenfold cross-validated classification accuracy on the training sample equaled AUC = 96%.

## Supplementary information

Supplementary Information 1.

## Data Availability

The datasets (excluding actual images) and code used to compute the results are available at https://osf.io/c58d3/.
