# Author Correction: Facial recognition technology can expose political orientation from naturalistic facial images

**DOI:** 10.1038/s41598-021-02785-z

**Published:** 2021-11-25

**Authors:** Michal Kosinski

**Affiliations:** Stanford University, Stanford, CA 94305 USA

Correction to: *Scientific Reports* https://doi.org/10.1038/s41598-020-79310-1, published online 11 January 2021

The original version of this Article contained an error in the Supplementary methods, under the subheading ‘Facebook sample’,

“The scales’ Cronbach’s α reliability equaled 84, 0.92, 0.93, 0.88, and 0.93 for openness, conscientiousness, extraversion, agreeableness, and neuroticism, respectively.”

now reads:

“The scales’ Cronbach’s α reliability equaled 0.84, 0.92, 0.93, 0.88, and 0.93 for openness, conscientiousness, extraversion, agreeableness, and neuroticism, respectively.”

The original Article has been corrected.

