# Supplementary material for: Facial recognition technology can expose political orientation from naturalistic facial images
Source: Sci Rep. 2021 Jan 11;11:100. doi: 10.1038/s41598-020-79310-1 (PMC7801376; doi:10.1038/s41598-020-79310-1)
Supplement: Supplementary file 1 — Supplementary Information 1. [file 41598_2020_79310_MOESM1_ESM.pdf]

Supplementary Information for

Facial recognition technology can expose political orientation from naturalistic facial images

Michal Kosinski (michalk@stanford.edu)

Supplementary Table S1. Accuracy afforded by facial features when predicting political orientation. All 95% confidence intervals are below 1% and are thus omitted.

		Dating Website			
		U.S.	Canada	UK	Facebook
Head pose	Yaw (absolute)	54%	53%	54%	51%
	Pitch	56%	57%	54%	51%
	Roll (absolute)	53%	53%	52%	52%
	Combined	58%	58%	57%	53%
Emotional expression	Neutral	53%	54%	51%	53%
	Sadness	50%	52%	50%	53%
	Disgust	51%	53%	50%	53%
	Anger	51%	52%	50%	54%
	Surprise	52%	50%	52%	55%
	Fear	51%	52%	50%	54%
	Happiness	50%	54%	51%	56%
	Combined	54%	56%	53%	57%
Other	Facial hair (males only)	51%	54%	62%	51%
	Glasses	50%	51%	51%	51%
	Sunglasses	51%	51%	52%	52%
	All facial cues	59%	60%	59%	59%

Supplementary Table S2. Intraclass correlation (ICC) between Face++ and two raters' estimates of head pose and emotional expressions based on a subset of 300 images from the Facebook sample. Two-way random, single-score (A,1) and two-way mixed, average-score (C,k) coefficients are reported.

	Facial Feature	ICC(A,1)	ICC(C,k)
Head pose	Yaw	.55	.71
	Pitch	.29	.45
	Roll	.14*	.25*
Emotional expression	Neutral	.82	.90
	Sadness	.28	.44
	Disgust	.51	.67
	Anger	.72	.83
	Surprise	.75	.86
	Fear	.52	.68
	Happiness	.86	.92

Note. The asterisks * indicate the p-value of <.01; other p-values are at the <.001 level.
